# Advances in Technologies in Crime Scene Investigation

**DOI:** 10.3390/diagnostics13203169

**Published:** 2023-10-10

**Authors:** Massimiliano Esposito, Francesco Sessa, Giuseppe Cocimano, Pietro Zuccarello, Salvatore Roccuzzo, Monica Salerno

**Affiliations:** 1 Faculty of Medicine and Surgery, “Kore” University of Enna, 94100 Enna, Italy; 2 Department of Medical, Surgical and Advanced Technologies “G.F. Ingrassia”, University of Catania, 95121 Catania, Italy; francesco.sessa@unict.it (F.S.); salvatore.roccuzzo.medicolegale@gmail.com (S.R.); monica.salerno@unict.it (M.S.); 3 Department of Mental and Physical Health and Preventive Medicine, University of Campania “Vanvitelli”, 80121 Napoli, Italy; peppecocimano1@gmail.com; 4 Laboratory of Forensic Toxicology, Department “G.F. Ingrassia”, University of Catania, 95124 Catania, Italy; pietrozuccarello85@gmail.com

**Keywords:** laser scanner, crime scene investigation, three-dimensional (3D) imaging, technological advances, forensic imaging

## Abstract

Crime scene investigation (CSI) is the complex act of reconstructing the dynamics that led to a crime and the circumstances of its perpetration. Crystallizing the CSI is a difficult task for the forensic pathologist; however, it is often requested by the public prosecutor and many judicial cases remain unsolved precisely for this reason. Recent years have seen an improvement in the ability of 3D scanning technology to obtain dense surface scans of large-scale spaces, for surveying, engineering, archaeology, and medical purposes such as forensics. The applications of this new technology are growing every day: forensic measurement of wounds in clinical reports, for example, reconstruction of traffic accidents, bullet trajectory studies in gunshot wounds, and 3D bloodstain pattern analysis. A retrospective analysis was conducted across all crime scene investigations performed by the forensic staff of the Department of Forensic Pathology of the University of Catania from January 2019 to June 2022. Inclusion criteria were the use of a laser scanner (LS), the use of a camera, a full investigative scene, and collection of circumstantial data thanks to the help of the judicial police. Cases in which the LS was not used were excluded. Out of 200 CSIs, 5 were included in the present study. In case number 1, the use of the LS made it possible to create a complete scale plan of the crime scene in a few hours, allowing a ship to be quickly returned to the judicial police officer. In case 2 (fall from a height), the LS clarified the suicidal intent of the deceased. In case number 3 it was possible to reconstruct a crime scene after many years. In case 4, the LS provided a great contribution in making a differential diagnosis between suicide and homicide. In case 5, the LS was fundamental for the COVID team in planning the study of COVID-19 pathways and areas within a hospital with the aim of reduction of nosocomial transmission. In conclusion, the use of the LS allowed the forensic staff to crystallize the investigative scene, making it a useful tool.

## 1. Introduction

CSI is a complex act that reconstructs the dynamics that determined a crime and the circumstances of its realization, with either a forensic pathologist or medical examiner as well as the official primarily responsible for the investigation [1,2]. To understand the nature of the possible crime and search for any traces of victims or the offender, the task of the coroner consists in research, identification, collection, and conservation of the death scene [3]. The main objectives of forensic medicine are not only to establish the cause and manner of death but also to record samples, to analyze the crime scene, and to identify the cadaver through an autopsy [4]. Traditional methods of documentation of a death scene include photography, sketches, and notes, electrostatic lifting, or casting, as well as field forms and video footage. An alternative, supplemental method of documenting transient evidence may be the three-dimensional (3D) LS [5].

The term “three-dimensional (3D) imaging” refers to techniques that can process accurate internal 3D data by obtaining volumetric pixels (or voxels) of the measured target [6]. The 3D imaging can generate high-resolution 3D digital images; LSs are available as both handheld and stationary units [7]. The last ten years have seen an improvement in the ability of 3D scanning technology to obtain dense surface scans of large-scale spaces for surveying, engineering, archaeology, and medical purposes such as forensic medicine [8,9]. The applications of this new technology are growing every day: forensic wound measurement in clinical reports, for example, traffic accident reconstruction, bullet trajectory studies in gunshot wounds, and 3D bloodstain pattern analysis [10,11,12]. The benefits of laser scanning over conventional photography are the reproducibility of the measurements from the 3D image and the possibility of manipulating the 3D images using software programs. The ambient lighting does not influence the performance of the scanner, and it can recognize missing elements from partial evidence [13]. The advantages of LSs over other 3D imaging technologies (such as computed tomography (CT) or magnetic resonance imaging (MRI)) include the relative cost of the equipment and maintenance, quick image generation, portability, and simple use with minimal training [14,15].

The present study reports a case series from different CSIs in which 3D imaging technologies using an LS were employed. Using 3D technology with the LS helped the forensic pathologist’s staff solve difficult court cases through thorough CSIs. The aim of this study is to demonstrate the importance and utility of 3D reconstruction in CSIs in assessing the manner and cause of death and all the data required in a judicial trial.

## 2. Materials and Methods

### 2.1. Case Selection

A retrospective analysis was conducted across all CSIs performed by the forensic staff of the Department of Forensic Pathology of the University of Catania from January 2019 to June 2022. Inclusion criteria were the use of an LS, the use of a camera, a full investigative scene, and collection of circumstantial data with the help of the judicial police. Cases in which the LS was not used were excluded.

### 2.2. LS Analysis

For the 3D documentation of the crime scene, the LEICA BLK360 LS (Wetzlar, Germany) was used. Close-range digital photogrammetry is a system used to precisely measure the 3D coordinates of points on an object. In this way, each object is detected by multiple points that are superimposed. The overlapping of the points serves to match the images taken from different angles so as to create a single clear and defined image, which is much more precise than those taken individually. The LEICA BLK360 first completes a rotation to measure the ambient light. Then, it makes a second rotation with several stops. At each stop, it catches a piece of a spherical image. Each piece is automatically stitched together to create a full-dome image. Next, the BLK360 executes the third rotation, scanning 360,000 laser points/sec, each with a unique 3D position. The scanner uses a simple algorithm, measuring the distances and angles of the rays that start from the laser and reflect on objects. In this way the 3D coordinates of all objects are calculated, composing a single three-dimensional cloud made up of millions of points. These point clouds allow the forensic pathologist to reconstruct a CSI accurately, with extreme precision, and in minute detail. The maximum range of the flight time used by the LS is 300 m [16]. The laser points are combined to replicate the objects, buildings, and land that surrounds them in the form of a point cloud. After scanning, all stitches are processed by the 3D design software (Autodesk software, Vers. 1 2018) [17].

In the case of closed spaces, the LS recognizes the whole point cloud, and the reconstruction is very simple. Attention must be paid to mirrors that must be shielded (with a sheet, for example); otherwise, the LS confuses the point cloud. Even in open places (for example, a forest), it is possible to create the point cloud; however, the staff must use targets placed on fixed points (for example, trees in a forest) in order to distinguish the various scans. This software can join all the points manually or automatically, superimposing them in order to create a unique image. At the end of the process, the CSI is available and usable; furthermore, photos can be extracted.

## 3. Results

Out of 200 CSIs, 5 were included in the present study.

### 3.1. Case 1

**Figure 1:**
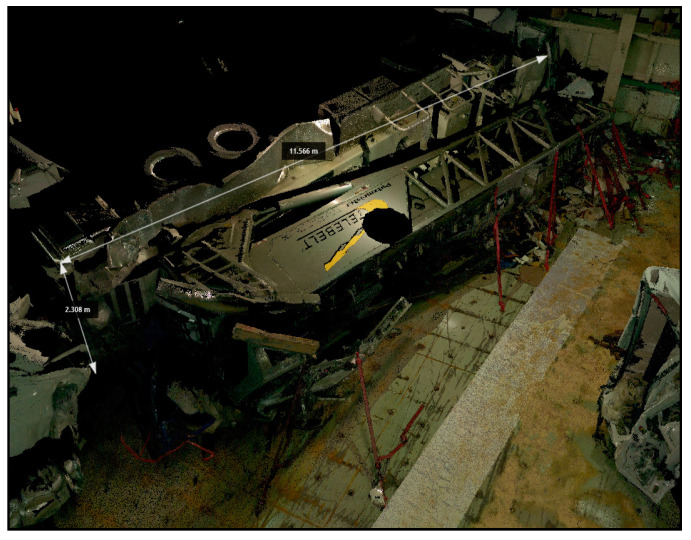
3D analyses of the CSI.

A 35-year-old man was found dead inside a “car carrier” depot on a cargo ship during an inspection on a flooded deck during a storm. The CSI was conducted through the LS that fixed the scene through a series of scans with an accuracy of 6 mm at 10 m. An inspection was performed of the truck that was damaged at the front and sides. An external examination of the cadaver was also performed. The presence of fractures at various body sites, excoriations, hematomas, and contusions was found. There was a perfect correspondence between the injuries found on the body and the impact against a solid and rigid surface of the truck. Thanks to the use of the LS, it was possible to have a complete reconstruction of the CSI (Figure 1).

### 3.2. Case 2

**Figure 2:**
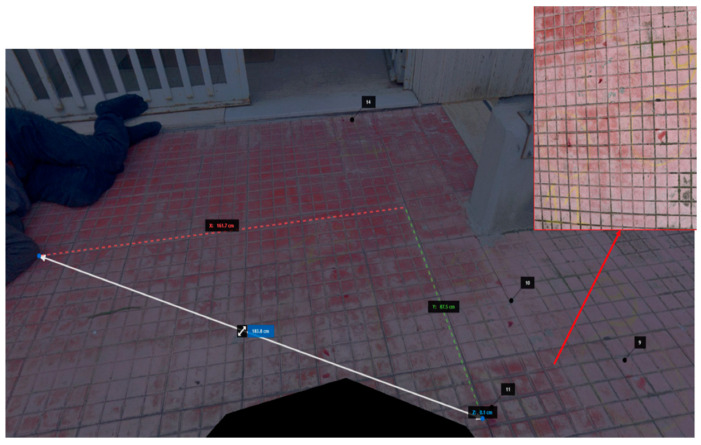
Case 2—3D reconstruction of the bloodstains.

A 34-year-old man suffering from anxiety and depression was found on the ground in the inner courtyard of his building. A tenant saw this man crawling in the courtyard, so he called the Territorial Emergency Service. The coroner saw that there were no signs of assault, just fractures of the chest bones. Through the LS, a study of the open spaces, internal courtyard, and balcony of the subject’s apartment was performed, as well as of the distances between the bloodstains and the corpse. Death was attributed to chest trauma from multiple fall-from-height trauma; these injuries were due to the violent impact of the body against the hard and rigid surfaces of the ground in the courtyard where the subject was found. These injuries are typical in cases of falls from heights; the circumstantial data, the results of the judicial investigation, and the external examination pointed to death by suicide from a fall from a height. In this case, the use of the LS was crucial in the study of the entire CSI, as it allowed the investigators to precisely examine the height of the precipice, the distance between the corpse and the bloodstains, the path of the corpse before dying, and therefore all the phases prior to death (Figure 2).

### 3.3. Case 3

**Figure 3:**
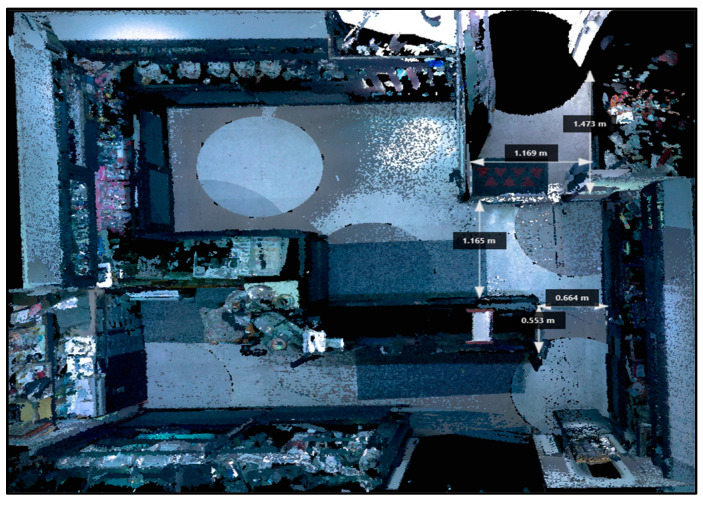
Case 3—3D reconstruction of the shop.

This CSI was carried out to re-evaluate an old murder case associated with a jewelry store robbery. The murder was carried out by the jeweler with a pistol shot at two thieves. To reconstruct the dynamics of the crime scene and to detect new data that could be analyzed in the process, the investigation was conducted using the LS. The total floor size of the jewelry shop was 450 × 365 cm. On the left wall near the entrance door, at a height of 92 cm from the floor, there was a metal hook painted white, in which a gray metal fragment was found, 2 cm in size. The measurements taken during the investigation of the crime scene, the back room, and the escape route were visible. The entire CSI reconstructed through the LS constituted evidence used during the judicial process (Figure 3).

### 3.4. Case 4

**Figure 4:**
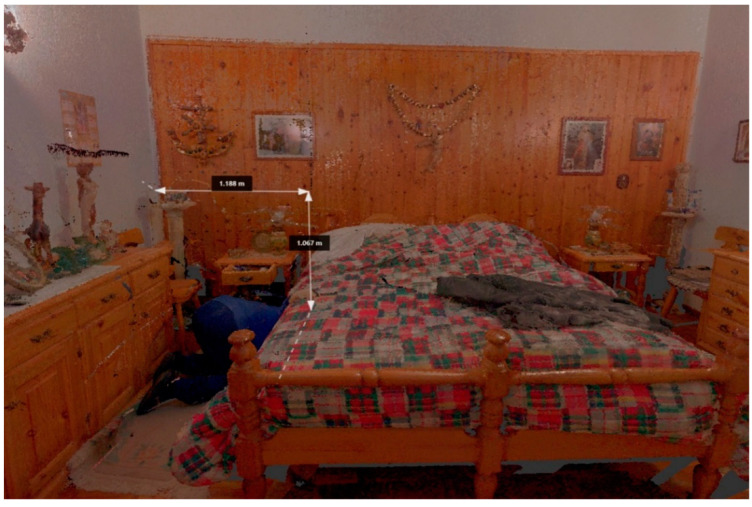
Case 4—3D reconstruction/measurements and position of the body.

An 86-year-old man suffering from depression, diabetes, leukemia, heart and respiratory disease was found dead in his apartment with a gunshot wound. The important aspect was to verify whether the death was due to a suicide or a homicide. During the CSI, the LS was used, which allowed the investigators to precisely analyze the distance between the ogive and the weapon, between the weapon and the corpse, and all the bloodstains present in the room. Thanks to the use of the LS at the crime scene and a thorough external examination performed by the forensic staff, it was concluded that the death was due to a suicide (Figure 4).

### 3.5. Case 5

**Figure 5:**
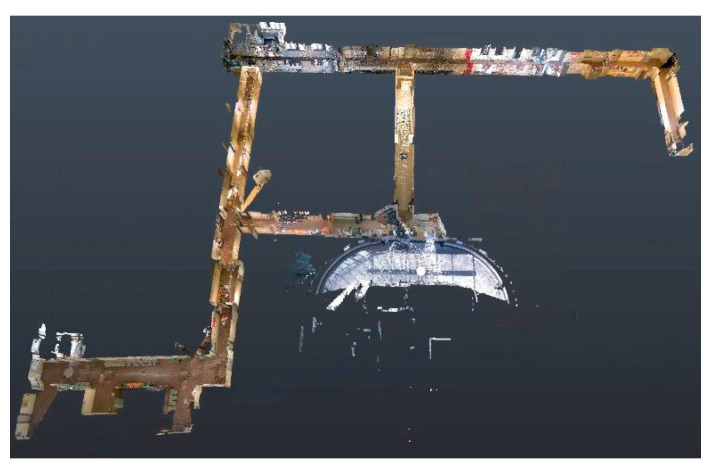
Case 5—3D reconstruction of the central block of the Hospital.

The forensic staff was involved in the reorganization of a Sicilian hospital during the COVID-19 pandemic, to plan the pathways of the healthcare personnel within the COVID-19 and non-COVID-19 areas of the hospital. Prior to the inspection, all personnel wore personal protective equipment (PPE) as per guidelines [18,19,20]. The staff proceeded with the analysis of the internal and external environments through the use of the LS, which made it possible to study and reorganize all the pathways of the COVID-19 areas, with the analysis of the PPE dressing areas of the healthcare personnel, the PPE removal areas, disinfection areas, and exit areas where healthcare personnel were “clean” (Figure 5).

## 4. Discussion

CSIs involve crucial and delicate work areas for the forensic pathologist due to the analysis of the body within the environmental context [21]. However, the contamination of a crime scene is often a sensitive issue carrying the risk of destruction of evidence [22,23,24]. The correct analysis and documentation of the crime scene is crucial in cases of mass disasters, where the dispersion of remains makes reconstruction of the events more difficult, and in cases of homicide [25]. The techniques of recording crime scene details have improved in the last few years with the introduction of modern 3D analysis systems such as LSs with the use of cameras [26]. Acquisition of 3D images can conserve the morphological and metric characteristics of the crime scene and reproduce the same measurements with high precision. In fact, LS technology has been applied since 2004 for accident and crime scenes. An LS is simple to use and the resulting 3D point cloud fusion allows an initial analysis of the 3D data [27,28,29,30]. The acquisition and the electronic storage of the crime scene data can be seen as virtual conservation of evidence. In fact, all the objects can be examined at any time in future analyses using a computer [31]. Using a laser scanner allows a fast, geometric reconstruction of complex scenes through dense point clouds. Finally, the images generated by 3D LSs are easy to manage (such as sharing with colleagues and consultants via email). Often the 2D projection level is not sufficient for forensic medicine and scientific analysis [32]. However, photogrammetry focuses better on the small details of the objects in the crime scene, and laser scanning gives a more comprehensive view of the geometry of the whole crime/accident scene. Both techniques can be used for capturing a scene just after a crime or a disaster has occurred and before the area is disturbed [33].

Buck et al. [27] affirmed that the 3D method is important for bloodstain pattern analysis in the determination of the trajectory of all blood spatters. The 3D crime scene documentation is able to recognize even the smallest bloodstains, and bloodstains on vertical, horizontal, and complex-shaped objects can be analyzed. In order to present the results in a copiable and measurable manner, the blood spatter directions, the trajectories, and the areas of origin of the bloodstains are rendered in the virtual true-to-scale 3D crime scene. The 3D laser scanning also offers a method of documenting and studying prehistoric human skeletons [9,34]. It can reproduce the scene in cases of road accidents and demonstrate the location of vehicles, objects, and involved persons at various points in time [35,36].

The present study reports five cases demonstrating the usefulness of an LS. In case number 1, the fixation of the crime scene was essential due to the impending repairs to the cargo ship. The use of the LS LEICA BLK360 allowed for the creation of a complete scale diagram of the crime scene in a few hours, allowing the vessel to be quickly returned to the judicial police officer. This reconstruction was based on a comparison of the 3D models using generated real data and mainly dealt with the geometric evaluation of the impact situation, with a subsequent reconstruction in 3D using specific Autodesk Recap Pro dedicated software (Vers. 1 2018). In cases number 2 and 4, the clarification of the suicidal rather than homicidal event was at the center of the investigation. The study of the crime scene’s open spaces, inner courtyard, and balcony apartment (case 2), as well as the distances between the bloodstains and the corpse (cases 2 and 4), and the close position of the gun to the corpse (case 4) made it possible to prove that there were no assault injuries on the body, in consideration of the circumstantial data and the results of the judicial investigation. In case number 3, the study of a crime scene that had changed over the years required special equipment suited to the “exposed” scenario. The LS Leica BLK360 made it possible to reconstruct the dynamics of the crime and, despite the obvious limitations of the case, it was possible to demonstrate the correspondence between the trajectory of the bullet and the escape route. Finally, in case 5, the LS Leica BLK360 was crucial for the COVID team in planning the study of COVID-19 pathways and areas within a hospital, thus reducing nosocomial transmission [37].

Using an LS, it is possible to create a database for CSIs that has a dual functionality [13]. It produces persistent storage of the data obtained from the crime scene, which is important for providing evidence in a court of law [8]. The second function is to create a new model for analysis of the scene data by crime scene investigators, which is important to aid the investigators by providing the data they require in a quick and easy to understand manner. It is also possible to retrieve an object from a database and modify it in various ways (for example, by segmenting it into smaller parts, deleting particular objects from it, etc.) [36].

Reconstructions can be presented in the form of written expertise or as an expert testimony in court or play-acted at the incident scene and photographed and/or filmed using accounts of either the defendants or the victims (should they have recovered), or witnesses [24].

This study can be summarized by the sentence “a picture says more than a thousand words”. It conveys a message more clearly and leaves less room for alternative explanations than a verbal report, in which the listener must visualize the narrative event in their mind and must constantly readjust as the report progresses. Graphic presentations, especially 3D models, are more usable and easier to explain, especially in the reporting of a CSI in front of a jury in a court.

## 5. Conclusions

In a CSI, the forensic pathologist or the medical examiner is the official primarily responsible for the complex act of reconstructing the dynamics of a crime scene [38]. The main objectives of forensic medicine are the freezing or fixation of the crime scene, findings, collection of traces, technical assessments, biological samples collection, identification of the cadaver (through autopsy), and records of the CSI [39]. Traditional methods of documentation of a death scene include photography, sketches, and notes, electrostatic lifting or casting, as well as field forms and video footage. An alternative, supplemental method of documenting transient evidence may be the 3D LS. The term “three-dimensional imaging” refers to techniques that can process accurate internal 3D data by obtaining volumetric pixels (or voxels) of the measured target. The 3D imaging is capable of generating high-resolution 3D digital images; LSs are available in both handheld and stationary units [40]. The present study collects for the first time a heterogeneous series of CSIs in which an LS was used, offering the scientific community many ideas for the use of this scientific technique not only in judicial cases, but also for public health purposes, such as the reorganization of hospital environments in the event of a pandemic or nosocomial infection.

## Data Availability

The authors confirm that the data supporting the findings of this study are available within the article.
